# Basic and clinical immunology – 3033: Structural analyses and allergencity predicition of sapodilla (Manikara zapota) acidic thaumatin-like protein by bioinformatics

**DOI:** 10.1186/1939-4551-6-S1-P208

**Published:** 2013-04-23

**Authors:** YP Venkatesh, HG Ashok Kumar

**Affiliations:** 1 Biochemistry & Nutrition, Csir-Central Food Technological Research Institute, Mysore, India

## Background

Oral allergy syndrome to sapodilla plum is caused by a 21 kD allergen existing as two isoforms: an acidic thaumatin-like protein (TLP-1) and a basic thaumatin-like protein (TLP-2). The composite sequence of TLP-1 comprising the N-terminal sequence 1-7 (A-T-F-D-V/I-V-N-) and the deduced sequence 8-207 (GenBank JN624813.1) was used for this analysis. The major objective of this study was to analyze the structural features and allergenicity prediction of TLP-1 by bioinformatics.

## Methods

Presence of transmembrane domain was analyzed by DAS server. Out of 2700 TLP sequences in the NCBI database, 870 are from Eudicotyledons comprising of 19 orders (sapodilla belongs to order Ericales). The deduced protein sequences were manually selected for only full length sequences resulting in 132 TLP sequences. For the purpose of uniformity, the mature sequence was trimmed to include the conserved N-terminus -N-X-C- (residues 7-9) and -F-C-X- at the C-terminus representing the 16^th^ cysteine. Phylogenetic tree was constructed using PHYLOGENY.FR. The 3D structure prediction of TLP-1 by homology modeling was carried out using HHPRED-MODELLER which generates a 3D model using a homologous template. The predicted model was compared with 8 plant TLP structures retrieved from PDB using RASMOL. The allergenicity prediction of TLP-1 was determined by ALGPRED, a server commonly used for allergenicity prediction of proteins.

## Results

Transmembrane helix domain is lacking in the mature TLP-1 sequence. TLP-1 shows structural similarity with other TLPs in having a central domain made up of antiparellel β-sheets supported by 2 domains on either side, one made up of only β-sheets and the other of β-sheets/α-helices; the entire structure is stabilized by 8 disulfides. Comparison of eudicot TLP sequences shows that TLPs comprise of isoforms of various pI values, and pI does not correlate with phylogenetic grouping. Phylogenetic tree shows that all TLPs can be broadly grouped into 2 clades each further grouping into 2 sub-clades. Allergenicity prediction of TLP-1 supports its experimental observation as an allergen (score, 1.76; threshold, −0.4).

## Conclusions

Sapodilla TLP-1 shows structural similarity to other plant TLPs in having identical 3 domains and 8 disulfide bonds. Based on allergenicity prediction, sapodilla TLP-1 is classified as an allergen.

